# Identification and characterization of a bacterial glutamic peptidase

**DOI:** 10.1186/1471-2091-11-47

**Published:** 2010-12-01

**Authors:** Kenneth Jensen, Peter R Østergaard, Reinhard Wilting, Søren F Lassen

**Affiliations:** 1 Novozymes A/S, 2880 Bagsværd, Denmark; 2 Plant Biochemistry Laboratory, Department of Plant Biology and Biotechnology, University of Copenhagen, 40 Thorvaldsensvej, DK-1871 Frederiksberg C, Copenhagen, Denmark

## Abstract

**Background:**

Glutamic peptidases, from the MEROPS family G1, are a distinct group of peptidases characterized by a catalytic dyad consisting of a glutamate and a glutamine residue, optimal activity at acidic pH and insensitivity towards the microbial derived protease inhibitor, pepstatin. Previously, only glutamic peptidases derived from filamentous fungi have been characterized.

**Results:**

We report the first characterization of a bacterial glutamic peptidase (pepG1), derived from the thermoacidophilic bacteria *Alicyclobacillus sp*. DSM 15716. The amino acid sequence identity between pepG1 and known fungal glutamic peptidases is only 24-30% but homology modeling, the presence of the glutamate/glutamine catalytic dyad and a number of highly conserved motifs strongly support the inclusion of pepG1 as a glutamic peptidase. Phylogenetic analysis places pepG1 and other putative bacterial and archaeal glutamic peptidases in a cluster separate from the fungal glutamic peptidases, indicating a divergent and independent evolution of bacterial and fungal glutamic peptidases. Purification of pepG1, heterologously expressed in *Bacillus subtilis*, was performed using hydrophobic interaction chromatography and ion exchange chromatography. The purified peptidase was characterized with respect to its physical properties. Temperature and pH optimums were found to be 60°C and pH 3-4, in agreement with the values observed for the fungal members of family G1. In addition, pepG1 was found to be pepstatin-insensitive, a characteristic signature of glutamic peptidases.

**Conclusions:**

Based on the obtained results, we suggest that pepG1 can be added to the MEROPS family G1 as the first characterized bacterial member.

## Background

Biotech industries are becoming more and more successful in providing enzymatic solutions to an ever increasing number of industrial processes. The combination of high-throughput screening methods and the low cost of full genome sequencing has greatly sped up the process of identifying and isolating genes that match the criteria for a given industrial process. Besides being able to catalyze the enzymatic reaction in the industrial process, the enzymes must also be able to survive the often harsh industrial conditions. One of the frequently required capabilities of an industrial enzyme is the ability to function at high temperatures in either an acidic or alkaline environment. Enzymes with such properties can either be designed *in silico *or by high-throughput screening of microorganisms. High-throughput screening is often the first choice because optimization of an existing enzyme to an industrial process is much simpler than *in silico *design. The high-throughput screening is performed at conditions made to mimic the industrial process in order to find existing enzymes already able to cope with the industrial environment. Again, these study enzymes are often found in microorganisms that are able to grow in extreme conditions. By taking advantage of the many published and freely available genomes, it is often possible to make an educated guess of which microorganisms would be interesting to screen for a certain enzyme. Screening of such microorganisms will often provide an extensive battery of enzymes optimized for the selected screening conditions.

A soil screening conducted by Novozymes A/S resulted in the discovery of a novel strain of *Alicyclobacillus *(WO 2005/066339). The thermoacidophilic bacterial strain was isolated at low pH (approx. 4.5) and high temperature (60°C). The genus was identified by 16 S rRNA analysis and showed a significant phylogenetic distance from the previously known strains of *Alicyclobacillus *(WO 2005/066339). The strain was deposited in the DMSZ bacteria collection as *Alicyclobacillus sp*. DSM 15716. A gene for a putative G1 peptidase was identified in a gene library screening for secreted enzymes using Transposon Assisted Signal Trapping (TAST) [1] of *Alicyclobacillus sp*. DSM 15716 (WO 2005/066339).

The peptidase showed significant sequence similarity to the peptidase family G1 [2], a family otherwise thought to be limited to the filamentous fungal species of the Ascomycota phylum [3]. The characterized proteins known to be part of the G1 family are aspergilloglutamic peptidase (AGP) from *Aspergillus niger *[4], scytalidoglutamic peptidase (SGP) from *Scytalidium lignicolum *[5], acid peptidases B and C (EapB and EapC) from *Cryphonectria parisitica *[6], *Penicillium marneffei *acid proteinase (PMAP-1) [7], *Talaromyces emersonii *glutamic peptidase 1 (TGP1) [8] and BcACP1 from *Botryotinia fuckeliana *[9].

Based on sequence homology, five bacterial and a single archaeal protein have been annotated as putative G1 peptidases at the MEROPS peptidase database, but biochemical characterizations have not been carried out to confirm their function [2]. Structural homology to fungal G1 peptidases and conservation of the catalytic residues indicate that pepG1 from *Alicyclobacillus sp*. DSM 15716 could be a bacterial G1 peptidase. In order to further examine its properties, we have amplified *pepG1 *from *Alicyclobacillus sp*. DSM 15716 genomic DNA and heterologously expressed it in *B. subtilis*. Following purification, pepG1 was characterized according to its physical properties, such as pH and temperature optimum and the effects of various protease inhibitors were determined. Based on these results, we suggest that pepG1 can be annotated as a G1 peptidase.

## Results and discussion

### Phylogenetic analysis of peptidase family G1

**Figure 1:**
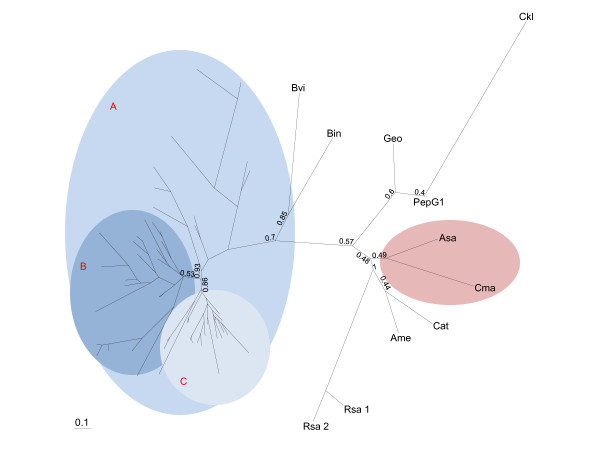
**Phylogenetic tree of peptidases from the MEROPS peptidase family G1**. The archaeal G1 peptidases are highlighted in rose. The fungal G1 peptidase cluster is highlighted in different shades of blue, and the major fungal clusters are labeled A, B and C. All annotated and putative family G1 peptidases (except for non-peptidase homologs) at the MEROPS peptidase database (version 9.1) were aligned using ClustalX version 2.0.11. The bootstrapped maximum likelihood tree was built using PhyML 3.0 aLRT [31] and visualized in TreeView [32]. The tree was bootstrapped with 100 iterations and bootstrap values are indicated on the figure. All GenBank accession numbers and detailed information on the members of each cluster can be found in Additional file 1 Table S1. **Asa**: [GenBank: YP_003816089] from *Acidilobus saccharovorans*; **Ame**: [GenBank: YP_003762485] from *Amycolatopsis mediterranei*; **Bin**: [GenBank: ACB95479] from *Beijerinckia indica*; **Bvi**: [GenBank: ABO59772] from *Burkholderia vietnamiensis*; **Cat**: [GenBank: YP_003114490] from *Catenulispora acidiphila*; **Ckl**: [GenBank: BAH07727] from *Clostridium kluyveri*; **Cma**: [GenBank: ABW02092] from *Caldivirga maquilingensis*; **Geo**: [GenBank: YP_003244752] from *Geobacillus sp*. Y412MC10; **PepG1**: [GenBank: HM011103] from *Alicyclobacillus sp*. DSM 15716; **Rsa_1**: [GenBank: ABY24309] from *Renibacterium salmoninarum*; **Rsa_2**: [GenBank: ABY21885] from *Renibacterium salmoninarum*.

Previously, only G1 peptidases derived from filamentous fungi have been characterized and the peptidase family G1 was thought to be limited to filamentous fungi - more precisely fungi from the Ascomycete phylum [3]. As the number of sequenced genomes increases, more and more hypothetical proteins are annotated based on sequence similarities to previously characterized proteins or protein signatures. The MEROPS peptidase database (version 9.1) [2] has assigned sixty-six open reading frames (ORFs) to family G1 with the majority being derived from Ascomycetes. Sixty of the ORFs are from Ascomycetes but six are supposedly non-peptidase homologs lacking one or both catalytic residues, thereby bringing the total number of Ascomycete peptidases down to fifty-four. The G1 peptidases are found in the following Ascomycete orders: Eurotiales, Pezizales, Sordariales, Leotiales, Diaporthales, Dothideales and Pleosporales, with the vast majority of G1 peptidases originating from the Eurotiales order (Additional file 1 Table S1). Of the remaining six ORFs in peptidase family G1, five are from bacteria and one is from archaea. In addition, blast searches at NCBI identified one more archaeal and three more bacterial G1 peptidase homologs. A bootstrapped unrooted maximum likelihood phylogenetic tree (disregarding the non-peptidase homologues) showed a clear distinction between Ascomycete and bacterial/archaeal pepG1 peptidases. The Ascomycete cluster A can be subdivided into two major clusters, termed B and C (Figure 1). All G1 peptidases derived from the *Eurotiales *and *Leotiales *orders had at least one paralog in each major cluster, as indicated in Additional file 1 Table S1. This strongly indicates that gene duplication took place before species differentiation in the *Eurotiales *and *Leotiales*. Each species, primarily in the *Eurotiales*, contains numerous paralogs [3] (i.e. seven in *Talaromyces stipitatus *and *P. marneffei*), which appears to be the result of extensive gene duplications within the species as many of the paralogs exhibit very high sequence similarity. The bootstrap confidence levels of the internal nodes of the Ascomycete clusters were in general above 0.7, indicating that the members of the different clusters are grouped correctly together. As expected, the bacterial and archaeal peptidase G1 orthologs were found to cluster separately from the Ascomycetes, supported by a bootstrap confidence value of 0.7 (Figure 1). The archaeal G1 peptidases were clustered together, but do not appear to be as divergent from the bacterial G1 peptidases as could be expected. A plausible explanation could be that "housekeeping genes" from archaea are bacterial in origin [10], although this assumption is still heavily debated. Another possibility could be that the introduction of G1 peptidases into archaea was facilitated by ancient horizontal gene transfer events. Low bootstrap values prevent deduction of the mutual relationship between the bacterial G1 peptidases from the generated maximum likelihood phylogenetic tree, except for the observation that bacterial G1 peptidases from Proteobacteria (Bin and Bvi) and Firmicutes (Ame, Cat, Ckl, Geo, pepG1 and Rsa 1+2) fall into two different clusters (Figure 1). Several attempts to improve the confidence levels of the bacterial/archaeal part of the phylogentic tree, including restricting the phylogenetic analysis to the most conserved regions of the sequences, were unsuccessful. On the other hand, no significant changes in the layout of the phylogenetic tree were observed by using only the most conserved regions, indicating that the present layout of the phylogenetic tree is acceptable. A possible explanation as to why the bootstrap values could not be improved may be due to the highly divergent amino acid sequences, illustrated by the low sequence homology between both the bacterial orthologs (25-35% sequence identity) and the bacterial and fungal orthologs (24-30% sequence identity).

### Catalytic residues and secondary structure of pepG1

Before the determination of the crystal structures of AGP and SGP, several attempts at elucidating the catalytic residues of G1 peptidases were carried out. Site-directed mutagenesis of conserved acidic residues was completed and the mutated enzymes were evaluated based on their catalytic activity. It is also known, that both AGP and SGP are expressed as precursor proteins which are autocatalytically processed into mature proteins in acidic conditions. By selecting both mutants unable to catalyze the conversion of precursor into mature protein, and those lacking peptidase activity, a glutamine (Q107 in SGP, Q133 in AGP) and a glutamate (E190 in SGP, E219 in AGP) were believed to be the active site residues of G1 peptidases [11-13]. The almost simultaneous publications of the near identical crystal structures of SGP and AGP verified the site-directed mutational studies and confirmed that the catalytic dyad in G1 peptidases consists of a glutamine and a glutamate residue [14,15].

**Figure 2:**
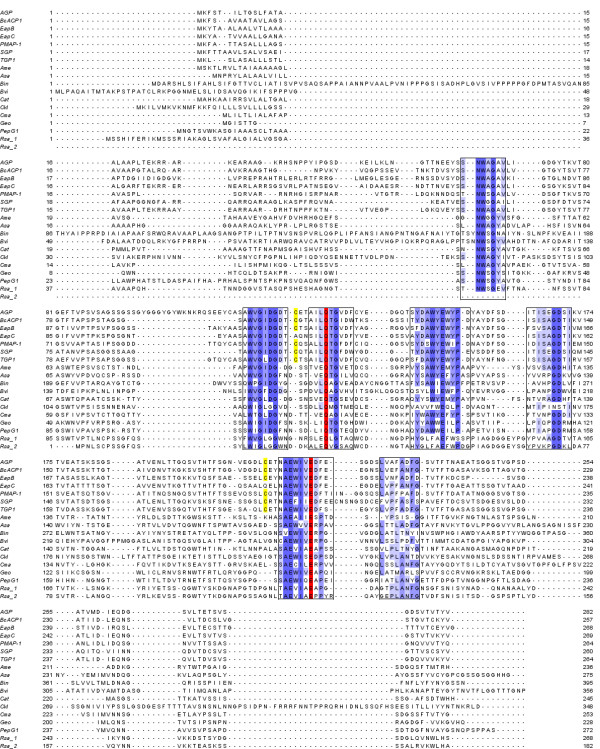
**Comparison of pepG1 with well-known family G1 peptidase and putative bacterial and archaeal members**. Full-length sequences including signal peptides were aligned using ClustalX version 2.0.11. The residues numbering for each peptidase is indicated. The seven highly conserved segments in all G1 peptidases are colored according to the percentage of the residues in each column that agrees with the consensus sequence. Only the residues that agree with the consensus residue for each column are colored. Dark blue means > 80%, blue > 60%, light blue > 40% and white < 40%. The catalytic dyad is colored red and the residues involved in a highly conserved disulfide bridge are shown in yellow. The fungal peptidases used for the alignment were aspergilloglutamic peptidase (AGP, [GenBank: P24665]) from *Aspergillus niger*, scytalidoglutamic peptidase (SGP, [GenBank: P15369]) from *Scytalidium lignicolum*, acid peptidases B and C (EapB [GenBank: Q00550] and EapC [GenBank: Q00551]) from *Cryphonectria parisitica*, *Penicillium marneffei *acid proteinase (PMAP-1, [GenBank: EEA28697]), BcACP1 ([GenBank: AAZ77775) from *Botryotinia fuckeliana *and *Talaromyces emersonii *glutamic peptidase 1 (TGP1, [GenBank: Q8X1C6]). The putative bacterial peptidases were [GenBank: YP_003762485] from *Amycolatopsis mediterranei *(Ame), [GenBank: ACB95479] from *Beijerinckia indica *(Bin), [GenBank: ABO59772] from *Burkholderia vietnamiensis *(Bvi), [GenBank: YP_003114490] from *Catenulispora acidiphila *(Cat), [GenBank: BAH07727] from *Clostridium kluyveri *(Ckl) and [GenBank: ABY24309], [GenBank: YP_003244752] from *Geobacillus sp*. (Geo), [GenBank: HM011103] from *Alicyclobacillus sp*. DSM 15716 (pepG1) and [GenBank: ABY21885] from *Renibacterium salmoninarum *(Rsa_1 and Rsa_2). The two archaeal peptidases were [GenBank: YP_003816089] from *Acidilobus saccharovorans *(Asa) and [GenBank: ABW02092] from *Caldivirga maquilingensis *(Cma).

An alignment of all G1 peptidases from the MEROPS database and pepG1 showed that the catalytic dyad was strictly conserved in pepG1 and all family G1 members, both characterized and putative. A simplified alignment showing the bacterial/archaeal members and the characterized fungal members are shown in Figure 2. The overall sequence similarities are, in general, low between the fungal and bacterial/archaeal peptidases, ranging from 24% to 30% amino acid identity. The crystal structure of SGP [14] revealed seven highly conserved motifs clustered on the upper β-sheet surrounding the active site and substrate-binding sites. The presence and high conservation of these motifs in both pepG1 and the other non-fungal putative members of G1 (Figure 2) strongly suggest that these enzymes are related members of fungal G1. Most mutations found in the motifs are conservative and therefore the general tertiary structure and function of the regions should be unaffected. SGP has three disulfide bridges, however not all are conserved in other G1 peptidases [14]. One is unique for SGP and of the two others, the most highly conserved one is located between C101 and C181 (Table 1) but is missing from EapC. The third disulfide bridge is specific to SGP and EapB and not found in any of the other fungal peptidases shown in the phylogenetic tree (Figure 1). None of the conserved cysteines are present in any of the bacterial or archaeal G1 homologs (Table 1). Disulfide bridge formation appears to have no direct effect on enzymatic activity but could result in more stable proteins as disulfide bridges are known to stabilize proteins at high temperatures [16].

**Table 1:** Protein signatures of known and hypothetical family G1 peptidases

Organism	Protein	Protein signatures				Active site residues		Disulphide bridge	
						
		IPR000250			IPR008985				
							
		PD18627	PR00977	PF001828					
Fungi	AGP	X	X	X	X	Q133	E219	C127	C210

Fungi	BcACP1	X	X	X	X	Q108	E194	C102	C185

Fungi	EapB	X	X	X	X	Q125	E210	C119	C201

Fungi	EapC	X	X	X	X	Q121	E206	A115	Q197

Fungi	PMAP-1	X	X	X	X	Q109	E196	C103	C187

Fungi	SGP	X	X	X	X	Q107	E190	C101	C181

Fungi	TGP1	X	X	X	X	Q116	E201	C110	C192

Bacteria	Ame	X		X	X	Q95	E176	C127	K167

Bacteria	Bin	X		X	X	Q226	E316		Q307

Bacteria	Bvi	X		X	X	Q173	E268		V259

Bacteria	Cat	X		X	X	Q99	E181		A172

Bacteria	Ckl	X		X	X	Q136	E224		I215

Bacteria	Geo	X		X	X	Q81	E162		

Bacteria	PepG1	X		X	X	Q117	E199		

Bacteria	Rsa_1			X	X	Q119	E208		G199

Bacteria	Rsa_2	X		X	X	Q31	E120		G111

Archaea	Asa	X		X	X	Q97	E183		D175

Archaea	Cma	X		X	X	Q92	E175		L167

**Figure 3:**
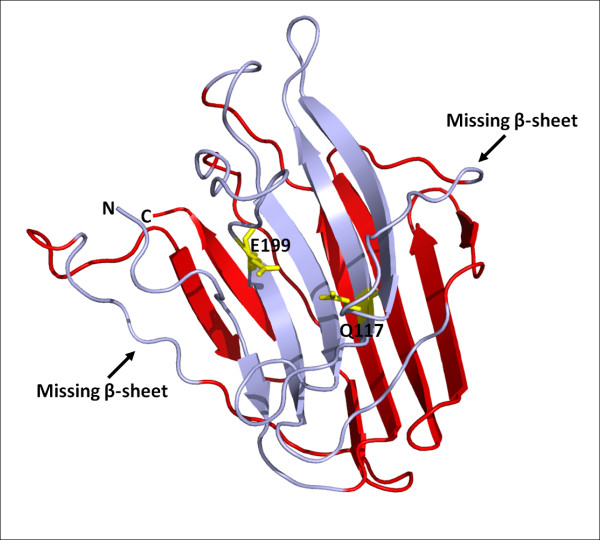
**Homology model of pepG1**. The model was generated using SWISS-MODEL [18] and visualized using PYMOL. The active site residues, Q117 and E199, are shown in yellow. The upper antiparallel β-sheet is light blue, and the lower β-sheet is red.

The structure determinations of AGP and SGP revealed a previously undescribed fold, comprised of a β-sandwich with two seven stranded antiparallel β-sheets [14,15]. Protein structure prediction of pepG1 using Phyre [17] identified AGP and SGP as the closest homologs to pepG1 and predicted that pepG1 had all fourteen β-sheets needed for the two seven stranded antiparallel β-sheet fold unique for G1 peptidases. No significant structural homology was found towards other proteins. To further examine the pepG1 structure, a three-dimensional model structure was generated using the SWISS-MODEL structure homology-modeling server [18]. A model structure encompassing residues 65-263 of pepG1 was obtained (Figure 3), corresponding to the mature pepG1 enzyme without the signal peptide. The structural template for the model structure of pepG1 was SGP [PDB: 2ifw], which has 23.5% sequence identity to pepG1. Stereochemistry of the backbone structure was evaluated by Ramachandran maps. Out of a total of 199 residues, only 12 were found in the disallowed and generously allowed regions. The PROCHECK [19,20] overall *g *factor, evaluating all torsion angles and bond lengths, was -0.5, indicating a good-quality model [21]. The two antiparallel β-sheet fold was present in the pepG1 homology model, but two of the β-sheets were missing from the upper section (Figure 3). The missing β-sheets are not believed to influence the catalytic activity of G1 peptidases. The active site residues, Q117 and E199, were found to be solvent exposed on the concave surface of the upper β-sheet. Both the orientations of the individual antiparallel β-sheets and the positions of active site residues in the pepG1 model are almost identical to the published structures of AGP and SGP [14,15]. The high structural similarity strongly supports that pepG1 is a G1 peptidase.

**Figure 4:**
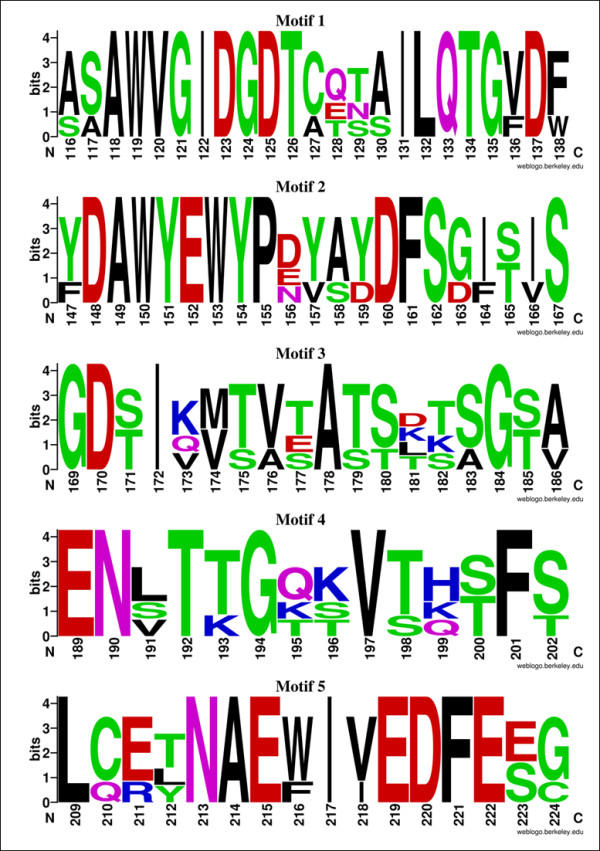
**WebLogo of the protein signature PR00977**. The sequence logo was constructed from the alignment of the four G1 peptidases AGP, SGP, EapB and EapC [22]. The letter size is proportional to the degree of amino acid conservation. The WebLogo was generated using WebLogo version 2.8.2 [34].

Sims et al [3] showed that G1 proteins carry several characteristic protein signatures. Investigation of the putative bacterial and archaeal G1 peptidases (Table 1) identified three out of four protein signatures. The missing protein signature, PR00977, is composed of five sequence motifs (Figure 4), of which four of them roughly correspond to the conserved motifs surrounding the active site [14] (Figure 2). A manual alignment of the PR00977 protein signatures to pepG1 showed that, although not all residues are conserved, the changes are mostly conservative. The PR00977 signature is based on an alignment of AGP, SGP, EapB and EapC[22]. The few sequences used for generating the PR00977 protein signature strongly restricts the allowed residue deviations (Figure 4) and would account for why the protein signature was not identified in the bacterial and archaeal G1 peptidases.

### Identification and expression of *pepG1*

The gene for a putative G1 peptidase was identified in a gene library screening for secreted enzymes using Transposon Assisted Signal Trapping [1] of *Alicyclobacillus sp*. DSM 15716 (WO 2005/066339). The gene encoding *pepG1 *was PCR amplified from genomic DNA of *Alicyclobacillus sp*. DSM 15716 and integrated by homologous recombination into the chromosome of *B. subtilis *MB1053. The signal peptide of pepG1 was replaced with a subtilisin-signal peptide for improved secretion in the *B. subtilis *host. SignalP cleavage site prediction for pepG1 was L_33_DA-SP [23]. Expression of pepG1 was tested in three different liquid medias at two different temperatures. Fermentation was continued for up to six days. The highest peptidase activity at pH 3.4, 50°C towards AZCL-collagen was observed after five days of growth in PS-I media. Degradation of AZCL-Collagen resulted in the formation of a blue halo. The diameter of the halo was used as a rough measurement of activity.

### Purification of pepG1

**Figure 5:**
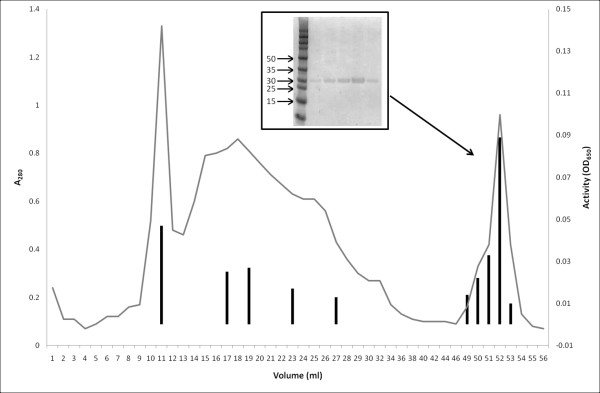
**Purification of pepG1**. Fractions 49-53 were subjected to SDS-page (insert). The size of the molecular marker is indicated on the left side of the SDS-page gel. The SDS-page gel was stained with Coomasie Brilliant Blue. The bars under the A_280 _trace indicate the activity of the individual fractions towards Protazyme OL (crosslinked and dyed collagen) at pH 4.0, 37°C.

Purification of pepG1 was performed as described in the material and methods section. A troublesome and unexpected high affinity of pepG1 to the ion exchange column used in the final purification step, resulted in only a partial elution of pepG1 (Figure 5). Fractions were analyzed for acid peptidase activity and as shown in Figure 5 pepG1 was eluted continuously in a broad peak and not a sharp peak as expected. Increased NaCl concentrations were required to elute the remaining pepG1 (fractions 49-56 in Figure 5). Fractions with acid peptidase activity were pooled and analyzed by SDS-PAGE. A single polypeptide band of 28 kDa was observed in the pooled fractions (Figure 5 insert), very similar to the molecular weights of about 22 kDa for AGP and SGP[24,25]. The total amount of purified protein was 226 mg/L. N-terminal sequencing was carried out on the purified protein and the following sequence (A_60_Q)N_62_FGWSASNWXGY, corresponding to the mature pepG1 peptidase, confirmed that the purified protein was pepG1.

### Characterization of pepG1

**Figure 6:**
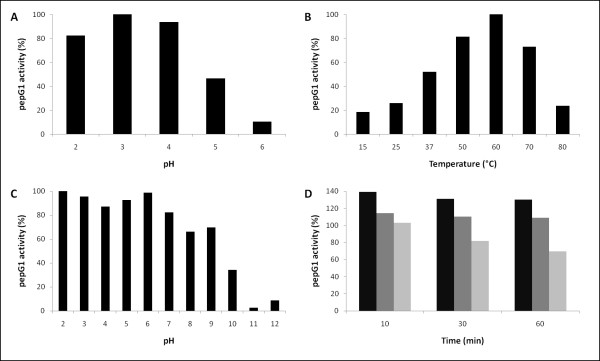
**Characterization of pepG1**. *A*. Effect of pH on pepG1 activity. The maximum activity at 37°C towards AZCL-collagen was obtained at a broad plateau around pH 3.0 and set at 100%. *B*. Determination of temperature optimum for pepG1. The maximum activity towards AZCL-collagen was observed at 60°C, pH 4.0 and set at 100%. *C*. pH stability of pepG1. pepG1 was diluted and incubated in assay buffer pH 2-12 for two hours at 37°C. pH was then adjusted to pH 4.0 and activity was measured at 37°C. *D*. Temperature stability of pepG1. pepG1 was incubated at 50°C (black), 60°C (grey) and 70°C (light grey) for up to one hour, cooled to 4°C on ice and assayed at 37°C, pH 4.0. Stability is measured relative to samples incubated on ice.

pepG1 exhibited peptidase activity towards AZCL-collagen, AZCL-casein and bovine serum albumin. AZCL-collagen was used for the characterization of pepG1 because of its higher stability at the experimental conditions (pH 2-12, 15-80°C) compared to AZCL-casein and bovine serum albumin. G1 peptidases are characterized by optimal enzymatic activity at low pH [2]. Peptidase activity for pepG1 was observed at pH values from 2.0 to 5.0, with a broad optimum pH range centered around pH 3.0 at 37°C (Figure 6a). The activity profile of pepG1 fits very well with the pH optimum of SGP, PMAP-1 and TGP1 [7,8,25]. 60°C, at pH 4.0, was found to be the optimal temperature for pepG1 proteolytic activity (Figure 6b). Temperature and pH optima fit the optimal growth conditions of the known thermophilic bacteria of the genus *Alicyclobacillus*, more precisely 35-60°C at pH 3.0-5.5 [26]. pepG1 was found to be a very stable protein, in regards to both the pH and temperature stability. Prolonged incubation at pH values of up to 6 had only minor effects on peptidase activity. Even at a pH of 9, the residual activity was still more than 50% (Figure 6c). Incubation at 70°C for up to one hour caused some reduction in pepG1 activity, but more than 60% activity was retained after one hour incubation at 70°C (Figure 6d). It was surprising that despite the lack of disulphide bridges, pepG1 had higher thermal stability than SGP. The single cysteine residue present in pepG1 is located in the N-terminal signal peptide and is removed from pepG1 after conversion of pepG1 into its mature form. SGP lost most of its activity after incubation at 70°C for fifteen minutes, despite its three disulfide bridges otherwise known to stabilize proteins at high temperatures [16]. An explanation for the higher stability of pepG1 could be due to the presence of a large number of electrostatic interactions and/or hydrophobic interactions, which are known to stabilize proteins at high temperatures.

### Effects of protease inhibitors and divalent cations on pepG1 activity

Many aspartic peptidases are strongly inhibited by the microbial derived inhibitor, pepstatin [27]. However, a hallmark feature of the G1 peptidases is their insensitivity towards pepstatin. Therefore, studies of pepG1 sensitivity towards four catalytic class-specific inhibitors, pepstatin, EDTA, PMSF and E-64 (L-trans-epoxysuccinyl-leucylamide-(4-guanidino)butane, N-(N-L-3-transcarboxyirane-2-carbonyl)-L-leucyl)-agmatine, were performed in order to further characterize pepG1. No significant inhibition was observed in the presence of the aspartic, serine and cysteine inhibitors, pepstatin, PMSF and E-64. Furthermore, pepG1 insensitivity towards EDTA suggests that metal ions are not required for activity (Table 2). Similar resistance to protease inhibitors are seen in fungal G1 peptidases [7,8,25,28]. Insensitivity towards the aspartic peptidase inhibitor pepstatin, is a characteristic feature of G1 peptidases and supports the assignment of pepG1 to the G1 family.

**Table 2:** Class-specific inhibitors effect on pepG1 activity

**pepG1 was incubated for 30 min with the below inhibitors at pH 4.0 (10 min, pH 4.5 for E-64). The remaining activity was assayed at 37°C**.			
**Inhibitor**	**Class-specific inhibitor**	**Concentration (mM)**	**Relative activity**

Pepstatin	Aspartic	0.005	0.92

EDTA	Metallo	10	0.93

PMSF	Serine	10	0.94

E-64	Cysteine	1	0.99


Relative activity is relative to the activity of pepG1 without inhibitor present.

Oda and Murao [25] showed that by incubating SGP for 30 min with the divalent cations Cu^2+ ^and Mn^2+^, a 50% increase in enzymatic activity occurred. Studies were performed with various divalent cations, including Cu^2+ ^and Mn^2+^, but only Cu^2+ ^had an effect on pepG1 enzymatic activity (Table 3).

**Table 3:** Effect of divalent cations on pepG1 activity

pepG1 was incubated for 30 min with the below cations at pH 4.0. The remaining activity was assayed at 37°C, pH 4.0		
**Cation**	**Concentration (mM)**	**Relative activity**

Cu^2+^	5	1.4

Fe^2+^	5	1.0

Zn^2+^	5	1.1

Mg^2+^	5	1.0

Mn^2+^	5	1.0

Ca^2+^	5	1.0

pepG1 activity in citric acid buffer pH 4.0 was set at 1.0

## Conclusions

Here we report the first characterization of a non-eukaryotic glutamic protease from the bacteria *Alicyclobacillus sp*. DSM 15716. Alignment of pepG1 with the known members of peptidase family G1 showed that the catalytic dyad, Q117 and E199 (pepG1 numbering) was conserved which indicates that the enzymatic mechanism is comparable to the fungal enzymes of this family. In addition, the crystal structure of SGP identified seven highly conserved motifs of the polypeptide chain clustered around the active and substrate-binding site of SGP [14]. These motifs are highly conserved in pepG1. Furthermore, protein structure prediction of pepG1 by Phyre [17] found SGP and AGP to be the closest homologs, which was supported by homology modeling of pepG1. Very high structural similarities were observed between the homology model of pepG1 and the crystal structures of AGP and SGP [14,15]. A number of protein signatures have been linked to G1 peptidases and three out of four are present in pepG1, despite the otherwise low sequence homology between pepG1 and the fungal G1 peptidases. The fourth signature could be identified by manual alignment and annotation of pepG1. The above bioinformatic studies of pepG1 clearly support the entry of pepG1 into the peptidase family G1.

To further validate the identity of pepG1, *pepG1 *was cloned into the expression host *B. subtilis*. Following expression and purification of pepG1, the pH and temperature optima of the peptidase and its stability were tested. In agreement with all G1 peptidases, pepG1 exhibited highest activity in acidic conditions. pepG1 was found to be resistant towards serine, cysteine, metallo and aspartic class-specific inhibitors, including pepstatin. Insensitivity to Pepstatin is a hallmark feature of all G1 peptidases.

Blast searches of the pepG1 sequence at NCBI identified several other putative bacterial G1 peptidases. If disregarding pepG1 homologs from related *Alicyclobacillus *species, new pepG1 homologs are found in the bacterias *Amycolatopsis mediterranei*, *Geobacillus sp*. and *Catenulispora acidiphila *along with archaeal homologs from *Acidilobus saccharovorans *and *Picrophilus torridus*. All of these homologs are between 40-50% identical to pepG1 and the active site residues, Q and E, that together form the catalytic dyad [14,15], are conserved in all homologs. The *in vivo *function of G1 peptidases in bacteria and archaea is presently unknown. The majority of the fungal species secreting G1 peptidases are pathogens [6-9], in which the peptidases are most likely used to facilitate host tissue penetration and colonization by degrading structural proteins of the plant cell wall [29]. The habitat of many of the microorganisms secreting G1 peptidases is soil or in some cases more extreme habitats, such as high temperature acidic environments. An obvious function could be scavenging as suggested by Fütterer et al, who sequenced and annotated the genome of the thermoacidophilic archaea, *Picrophilus torridus *[30].

The characterization of pepG1 presented in this manuscript along with the demonstrated presence of putative G1 peptidase homologs in an increasing number of non-fungal organisms strongly suggests that the non-fungal peptidase G1 homologs assigned to the MEROPS peptidase family G1 are correctly annotated.

## Methods

### Bioinformatics

All annotated and putative family G1 peptidases (except the non-peptidase homologues) in the MEROPS peptidase database (version 9.1) [2] as well as putative G1 peptidases identified by blast search at NCBI were aligned using ClustalX version 2.0.11. Bootstrapped maximum likelihood (100 iterations) phylogenetic tree was generated using ClustalX and PhyML 3.0 aLRT http://www.phylogeny.fr[31], respectively. Phylogenetic trees were visualized using TreeView http://taxonomy.zoology.gla.ac.uk/rod/treeview.html[32]. Protein signatures in the bacterial and archaeal peptidases were identified using InterProScan [22] and ProDom [33]. Sequence logo of the protein signature PR00977 was visualized using WebLogo version 2.8.2 [34]. A model spanning residues 65-263 of pepG1 was generated using SWISS MODEL [18]. The model structure was based on the PBD-file 2ifw and subsequently verified using PROCHECK [19,20] and Ramachandran maps generated by PDBSum [35]. PYMOL http://www.pymol.org was used for visualizing the model structure of pepG1.

### Bacterial strain and culture conditions

*Alicyclobacillus sp*. DSM 15716 was grown on ATBA-1 agar pH 4.5 (400 ml of 0.625% Tryptone (Difco), 0.625% amylopectin (ICN) and 2.5% agar, granulated (Difco) mixed with 100 ml of 0.1% ammonium sulfate, 0.25% magnesium sulfate, 0.125% calcium chloride and 1.5% potassium dihydrogen phosphate) at 60°C overnight.

### Cloning of *pepG1 *into *Bacillus subtilis *MB1053

The gene encoding *pepG1 *was amplified by PCR from genomic DNA of *Alicyclobacillus sp*. DSM 15716 and integrated by homologous recombination in *B. subtilis *MB1053 (*amyE*, *apr*, *npr*), in which the native subtilisin peptidase has been knocked out (WO03/0956658). Homologous recombination was done using an integration cassette consisting of two regions (with homology to the integration site on the *B. subtilis *genome) that together flanked *pepG1 *under control of a triple promoter. The triple promoter system consists of the promoters from *Bacillus licheniformis *alpha-amylase gene (*amyL*), *Bacillus amyloliquefaciens *alpha-amylase gene (*amyQ*), and the *Bacillus thuringiensis cryIIIA *promoter [36]. The two flanking regions were amplified from a modified *B. subtilis *MB1053 strain in which the Spectinomycin gene has been replaced with a marker gene encoding Chloramphenicol and a gene encoding the subtilisin protease, SAVINASE™. The 5'-flanking region covers the *yfmD *gene to the SAVINASE™-signal-peptide (included) and introduces an overhang to *pepG1*. The 3'-flanking region located downstreams from the SAVINASE™gene covers *Pel(end)-yflS-citS *and introduces an overhang to the 3'-end of *pepG1*. The *B. subtilis *MB1053 cell strain was made competent according to Yasbin et al [37].

### Nucleotide sequence analysis

The DNA sequences from both strands were determined with the BigDye Terminator v3.1 Cycle Sequencing Kit (Perkin Elmer) and Applied Biosystems 3730 XL DNA analyzer according to manufacturer's instructions.

### Selection of constructs for purification

The construct was grown in three different liquid media, PS-1 (10% sucrose (Danisco), 4% Soymeal (Cargill), 1% Na_2_HPO_4_•12H_2_O, 0.5% CaCO_3 _and 0.01% Dowfax 63N10), Cal18 (4% Yeast extract (Difco), 0.13% MgSO_4_•7H_2_O, 5% Maltodextrin (Roquette), 2% Na_2_HPO_4_•12H_2_O, 0.67% Na_2_MoO_4 _Trace metal solution and 0.01% Dowfax 63N10) and SK-1 M (4% Sodium Caseinate (MD-Food), 20% Maltodextrin, 5% Soybean meal and 0.01% Dowfax 63N10), all supplemented with 6 mg/L chloramphenicol. Fermentations were performed on rotary shaking tables in 500 ml baffled Erlenmeyer flasks each containing 100 ml liquid media at 37°C and 30°C. Samples were taken at day 2, 3 and 4 from Cal18 media and day 4, 5 and 6 from PS-1 and SK-1 M and analyzed for activity. The activity was determined by a spot test of 20 μl supernatant in 1% agarose plates at pH 3.4 with 0.1% AZCL-Collagen. The plates were incubated at 50°C over-night and activity was visible as a blue halo around the spots.

### Fermentation and purification of *A. sp*. pepG1

Fermentation of *B. subtilis *expression clone was performed on a rotary shaking table in 500 ml baffled Erlenmeyer flasks each containing 100 ml PS-1 media supplemented with 6 mg/L chloramphenicol. The clone was grown for five days at 37°C. Culture broth was centrifuged (20000 × g, 20 min) and the supernatant was filtered through a Seitz EKS filter plate. The EKS filtrate was adjusted to a pH of 4.0 with citric acid and heated to 70°C with continued stirring in a water bath. The solution was immediately placed on ice after the temperature reached 70°C. The precipitate was removed by a second filtration using a Seitz EKS filter plate. (NH_4_)_2_SO_4 _was added to a final concentration of 1.6 M and the pool was applied to a Butyl-Toyopearl 650 S column (bed volume 30 ml) equilibrated in 20 mM CH_3_COOH/NaOH, 1.6 M (NH_4_)_2_SO_4_, pH 4.5. After washing the column extensively with the equilibration buffer, protein elution was done with a linear gradient between the equilibration buffer and 20 mM CH_3_COOH/NaOH, pH 4.5 with 25% 2-propanol. Fractions from the column were analyzed for protease activity at pH 4.0, 37°C and fractions with activity were pooled. The pooled fractions were transferred to 20 mM CH_3_COOH/NaOH, pH 5.5 on a G25 sephadex column and applied to a Source 30Q column (bed volume of 40 ml) equilibrated in the same buffer. After washing the column thoroughly with the equilibration buffer, the protease was eluted with a linear NaCl gradient (0 to 0.5 M) in the same buffer. Fractions from the column were analyzed for protease activity (pH 4.0, 37°C). An additional elution with 1.0 M NaCl, 20 mM CH_3_COOH/NaOH, pH 5.5 was performed in order to release the remaining pepG1 from the column and fractions with activity were pooled. The slightly colored pool was treated with 1% (w/v) activated charcoal for 5 minutes and then passed through a 0.45 μm filter. The purity of the filtrate was analyzed by SDS-page and protein concentrations determined using Bradford protein assay.

### N-terminal sequencing

Automated Edman degradation of purified pepG1 was accomplished with a Perkin-Elmer ABI 494HT sequencer with online microbore phenylthiohydantoin-amino acid detection.

### Enzyme assays

Protease enzyme activity was assayed using Protazyme OL (crosslinked and dyed collagen from Megazyme). A Protazyme OL tablet was suspended in 2.0 ml 0.01% Triton X-100 by gentle stirring. 500 μl of the Protazyme suspension and 500 μl assay buffer (100 mM succinic acid, 100 mM HEPES, 100 mM CHES, 100 mM CABS, 1 mM CaCl_2_, 150 mM KCl, 0.01% Triton X-100 pH 4.0) were mixed in an Eppendorf tube and placed on ice. 20 μl protease sample was added and the assay initiated by transferring the Eppendorf tube to an Eppendorf thermomixer set at the assay temperature. The tube was incubated for 15 min on the Eppendorf thermomixer at its highest shaking rate (1400 rpm) and the reaction was stopped by transferring the tube back into the ice bath. The samples were then centrifuged in an icecold centrifuge for 3 min at 20,000 g and 200 μL supernatant was measured at OD_650_. A buffer blind without enzyme was included in the assay. OD_650_(Enzyme) - OD_650_(buffer blind) was used to express enzyme activity.

The above assay was used to determine the pH and temperature effect on activity, pH stability and temperature stability. pepG1 temperature stability was determined by incubating the enzyme at 50°C, 60°C and 70°C. Samples were taken after 10, 30 and 60 minutes of incubation, cooled on ice and assayed at 37°C, pH 4.0 in order to determine residual activity. pH stability was determined by diluting pepG1 5× in assay buffer pH 2-12 (total volume 100 μl) followed by incubation at 37°C for 2 hours. After incubation, 440 μl assay buffer pH 4.0 was added and assay was performed as described above. pH of the assay buffer was adjusted by addition of either NaOH or HCl.

### Effect of divalent metal ions on *A. sp*. pepG1 activity

Purified *A. sp*. pepG1 protease (20 μl) was incubated for 30 min in 500 μl citric acid buffer pH 4.0 (33 mM citric acid/17 mM sodium citrate and 0.01% Triton X-100) containing 5 mM concentrations of divalent ions. These samples were then assayed for activity with Protazyme OL suspended in 500 μl of citric acid buffer pH 4.0 containing a 5 mM concentration of the divalent ion at 37°C for 15 min.

### Inhibitor studies on *A. sp*. pepG1

Purified *A. sp*. pepG1 protease (20 μl) was incubated with the inhibitors, Pepstatin, EDTA and PMSF, for 30 min in 500 μl universal buffer pH 4.0. E-64 treatment of pepG1 was carried out for 10 min in 20 mM CH3COOH/NaOH, 1 mM CaCl2, pH 4.5. All samples were assayed for residual activity with Protazyme OL tablets at pH 4.0 (pH 4.5 for E-64), 37°C with the inhibitors present at the same concentrations as during the incubation.

### Accession numbers

Family G1 peptidase *pepG1 *from *Alicyclobacillus sp*. DSM 15716 [GenBank: HM011103].

## Authors' contributions

KJ was involved in all of the experimental and theoretical work and drafted the manuscript. PRØ participated in the enzyme purification and characterization and has commented on the manuscript. RW carried out the initial identification and work on pepG1 and has commented on the manuscript. SFL participated in the cloning, helped with coordination of the experimental work, was supervisor for KJ and helped to draft the manuscript. All authors have read and approved the final manuscript.

## Supplementary Material

Additional file 1 **Glutamic peptidases from MEROPS family G1**. A schematic overview of all glutamic peptidases including accession numbers. Click here for file
